# Cost-utility analysis of transitional care services for older inpatients with chronic obstructive pulmonary disease (COPD) in Korea

**DOI:** 10.1186/s12962-024-00526-3

**Published:** 2024-03-02

**Authors:** Yu Seong Hwang, Woo Jin Kim, Tae Hyun Kim, Yukyung Park, Su Mi Jung, Heui Sug Jo

**Affiliations:** 1 Department of Health Policy and Management, School of Medicine, Kangwon National University, Chuncheon-si, Gangwon State Republic of Korea; 2 Department of Internal Medicine and Environmental Health Center, School of Medicine , Kangwon National University , Chuncheon-si, Gangwon State Republic of Korea; 3 Department of Healthcare Management, Yonsei University Graduate School of Public Health, Seoul, Republic of Korea; 4 Department of Preventive Medicine, Kangwon National University Hospital, Chuncheon-si, Gangwon State Republic of Korea; 5 Team of Public Medical Policy Development, Gangwon State Research Institute for People’s Health, Chuncheon-si, Gangwon State Republic of Korea

**Keywords:** Chronic obstructive pulmonary disease, Transitional care service, Economic evaluation, Markov model, Cost-effectiveness analysis

## Abstract

**Background:**

Chronic obstructive pulmonary disease (COPD) is associated with a high readmission rate and poses a significant disease burden. South Korea initiated pilot projects on transitional care services (TCS) to reduce readmissions. However, evidence from cost-effectiveness analyses remains undiscovered. This study aimed to evaluate the cost-effectiveness of TCS in patients with COPD from the healthcare system’ perspective.

**Method:**

A cost-utility analysis was conducted using a Markov model containing six components of possible medical use after discharge. Transition probabilities and medical costs were extracted from the National Health Insurance Service Senior Cohort (NHIS-SC), and utility data were obtained from published literature. Sensitivity analyses were performed to test the robustness of the results.

**Results:**

Conducting TCS produced an incremental quality-adjusted life years gain of 0.231, 0.275, 0.296 for those in their 60s, 70s, and 80s, respectively, and cost savings of $225.16, $1668, and $2251.64 for those in their 60s, 70s, and 80s, respectively, per patient over a 10-year time horizon. The deterministic sensitivity analysis indicated that the TCS cost and the cost of readmission by other diseases immensely impact the results. The probabilistic sensitivity analyses showed that the probability that the incremental cost-effectiveness ratio is below $23,050 was over 85%, 93%, and 97% for those in the 60s, 70s, and 80s, respectively.

**Conclusions:**

TCS was the dominant option compared to usual care. However, it is advantageous to the healthcare budget preferentially consider patients aged over 70 years with severe TCS symptoms. In addition, it is essential to include the management of underlying comorbidities in TCS intervention.

**Trial registration:**

Clinical Research Information Service (CRIS), KCT0007937. Registered on 24 November 2022

**Supplementary Information:**

The online version contains supplementary material available at 10.1186/s12962-024-00526-3.

## Introduction

Chronic obstructive pulmonary disease (COPD) is a disease characterized by airflow limitation that does not completely recover and causes respiratory symptoms by damaging the airways and alveoli through smoking or indoor and outdoor dust/gas [1]. It has a high worldwide prevalence of approximately 11.7% [2] and was ranked the third leading cause of death worldwide in 2019 [3]. Furthermore, it is the ninth leading cause of death in Korea [4].

The readmission rate in patients with COPD is higher than that in patients with other diseases [5–6]. According to a meta-analysis, readmission rates for acute exacerbation reached 11% within one month, 17% within three months, and 37% within one year [7]. According to Korean statistics, the risk-standardized readmission rate for COPD in 2017 was 12.7%, the highest among the disease groups evaluated [8], and the unplanned readmission rate in 2020 was 16.9%, the second highest among the diseases evaluated [9]. Therefore, it is necessary to develop measures to reduce the incidence and provide continuous treatment and management strategies.

The COPD Clinical Guidelines recommend providing discharged patients with additional guidance, such as (a) disease education, (b) medication optimization, (c) inhaler-use education, (d) comorbidity assessment and management, (e) respiratory rehabilitation, and (f) monitoring and follow-up [1]. In addition, since smoking can directly affect COPD exacerbation by reducing lung function [10], smoking cessation is strongly recommended regardless of COPD severity [10]. However, providing sufficient medical education to patients is challenging owing to limited consultation hours and staff [11].

Transitional care service (TCS) refers to a bundle of services in which a professional nurse (a) establishes a comprehensive treatment plan for each patient, (b) conducts self-management education, and (c) provides ongoing management through phone calls or home visits [12–14] to the patients when the treatment environment changes, such as discharge from the hospital and going home.

The results are mixed regarding the effectiveness of TCS. Studies advocating for the effect of TCS have shown that when patients with COPD are treated with transitional care, the risk of being readmitted for COPD within six months is reduced by about half [15], and the patient’s cognitive and emotional state is enhanced, as well as the quality of life of the patient, family, and caregivers [16]. In addition, studies on the economic evaluation of TCS for patients with COPD discovered that patients who were included the discharge patient management program spent less on medical expenses than those who did not [17–18]. However, some studies have reported that interventions in discharged patient management programs do not affect readmission or mortality after discharge or exceed the willingness-to-pay (WTP) threshold [19–20].

After the announcement of the Comprehensive Plan for Public Health and Medical Development (October 2018) [21], the Korean government funded national university hospitals and local medical centers to conduct a project to establish a care plan for discharged patients and connect them with local community services. However, an economic evaluation thereof is yet to be conducted.

Considering the characteristics of the Korean medical market, such as the nationwide medical insurance system, fee-for-service system, low proportion of public hospitals, and high indemnity insurance subscription rate, it is necessary to develop an economic evaluation model suitable for the domestic situation.

Since South Korea has the National Health Insurance Service (NHIS) that provides healthcare coverage to all citizens, claims data from the NHIS can track a patient’s medical use, such as readmission, emergency room visits, or outpatient visits, without omission. Therefore, using claims data from the NHIS has the advantage of evaluating the cost-effectiveness of TCS.

In previous studies that evaluated the cost-effectiveness of discharge management for COPD patients, only the frequencies of COPD exacerbations were assumed as a severity (very severe, severe, moderate) and considered as transition states [22–23]. However, possible medical use after discharge needs to be included in the transition states since TCS aims to reduce unnecessary medical use by providing personalized interventions. In addition, the relative risk (RR), defined as a risk ratio of probability for being in a transition state for the intervention group compared to the usual care group, a single RR has been applied in traditional cost-effectiveness analysis. However, since we suppose diverse medical use states, we must apply RRs for every transition state. Nonetheless, to our knowledge, no study applied possible medical use as transition states nor RRs for the transition states. Therefore, we developed a new economic evaluation model that applies the conditions above to achieve the following research objectives:

- To develop a model to evaluate the cost-effectiveness of TCS in older patients with COPD in Korea.
- To identify how much cost and how effective it will be to provide TCS from the perspective of the healthcare system.
- To identify which age group is suitable for applying TCS.

## Methods

### Study design

The Markov model simulates medical use over a time horizon of 10 years with a cycle length of three months. In COPD clinical trials, 90 days have been reported as sufficient time to evaluate the intervention effect and prognosis thereof [24–26]. In addition, considering that COPD is a chronic disease, 10 years was set as the analysis period, giving a total of 40 cycles.

**Fig. 1:**
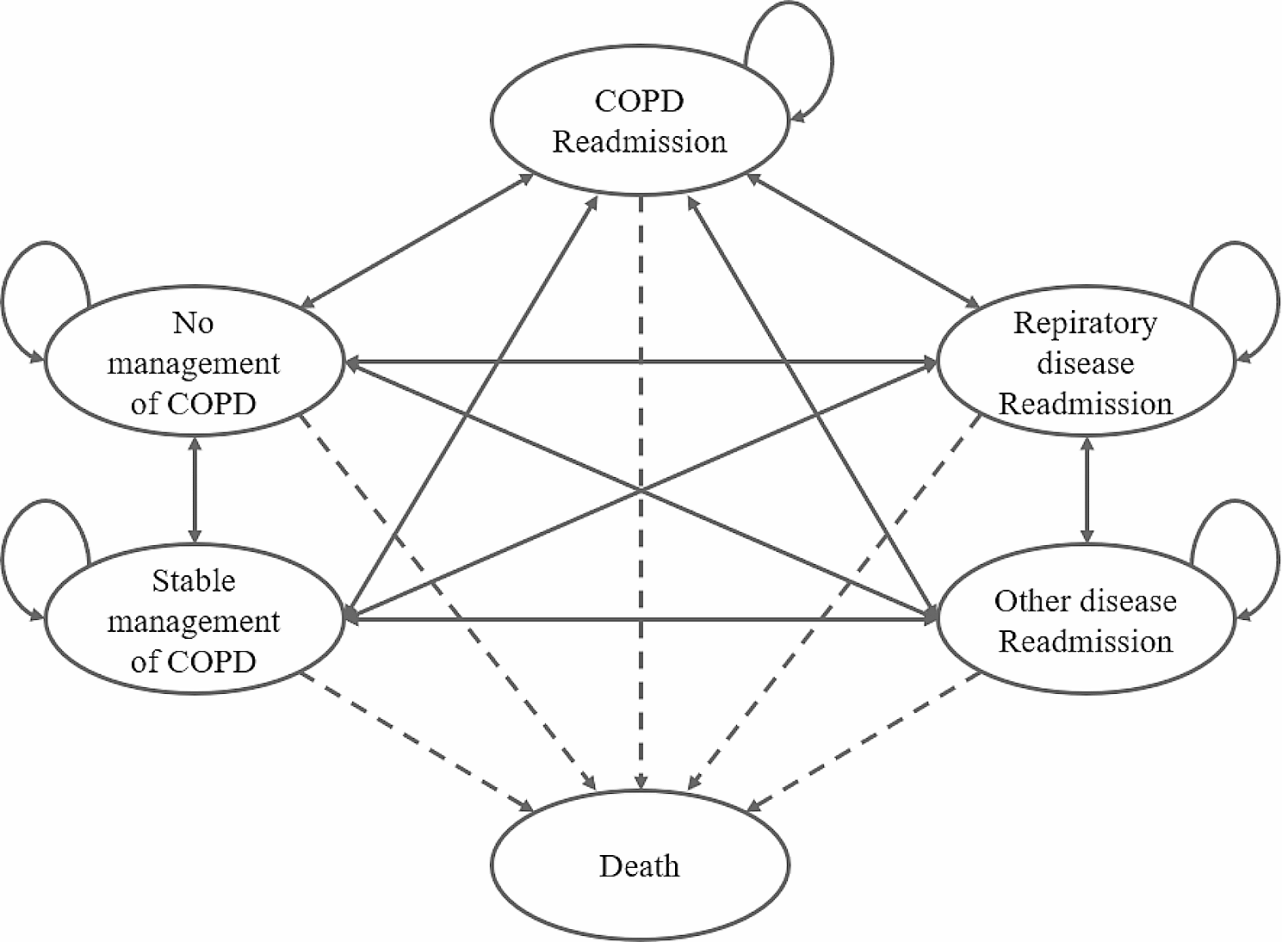
Markov model of six medical use states applied for the cost-effectiveness analysis

The model structure contains six transition states that patients with COPD may experience after their discharge. If a patient had a record of outpatient visits for COPD within 90 days, we assumed that the patient was in the “stable management of COPD” state. This is because patients with COPD must ordinarily see their doctors within four weeks after discharge and receive a new inhaler drug from the doctor’s prescription, as the inhaler drug doses are 30 or 60 days in volume. However, if a patient did not visit a doctor within 90 days, we assumed that the patient was in the “no management of COPD” state. Readmissions were divided according to their cause, including COPD, respiratory disease, and other diseases. After one cycle, the patients either remained in their state or moved to another state. <Figure 1>.

The transition probability readmission rate and medical costs for patients with COPD by disease were analyzed using Senior Cohort, version 2.0, with claims data provided by the NHIS <Table 1>. The Senior Cohort is composed by sampling 8% of the population, stratified random sampling by gender, age, insurance premium quintile, and region. The period of analysis was from 2009 to 2019, and patients with COPD who visited outpatient clinics between 2007 and 2008 were considered to have COPD already and were excluded. Patients included were hospitalized with J43 (emphysema) and J44 (other chronic obstructive pulmonary diseases) as the main illnesses, based on the Korean Standard Classification of Diseases (KCD Ver.7.0) [27]. Given the inherent structure of claims data, billing was conducted on a monthly basis, and records were maintained for hospital transfers or consultations with other departments during hospitalization. Consequently, operational definitions were employed to provide clarity in identifying readmissions. A case was considered a singular hospitalization if it satisfied all three criteria: (a) readmission occurred within one day following discharge, (b) the same healthcare institution identifier was utilized, and (c) the primary diagnosis code remained consistent. Patients were excluded if consultations with other departments occurred during their hospitalization, given the unavailability of recorded medication days. SAS 9.4 (Armonk, NY, USA) was used for the analysis.

**Table 1:** Transition probabilities by transition states and age cohort

Transition states		60s		70s		80s	
		*n*	(%)	*n*	(%)	*n*	(%)
Not readmitted	Stable management of COPD	548	34.73	892	30.90	414	24.34
	Not managing COPD	519	32.89	875	30.31	530	31.16
Readmitted	COPD	118	7.48	240	8.31	175	10.29
	respiratory diseases	108	6.84	245	8.49	172	10.11
	other diseases	285	18.06	635	22.00	410	24.10
Total		1,578	100	2,887	100	1,701	100

Relative risk is a value that represents the relative risk of belonging to a transition state that is harmful to health in the experimental group who participate in the TCS, compared to the control group. The results of previous studies [28–29] were used to determine the relative risk of readmission in TCS. According to a systematic literature review study that analyzed the effects of transitional medical services on patients with COPD, the risk of readmission due to COPD for patients who had implemented the discharge management program compared to patients who had not implemented the discharge management program was 0.599 (95% confidence index (CI) 0.421–0.852). The relative risk rate for all types of readmissions was 0.720 (95% CI 0.531–0.978) [28]. Norwood (2013) proposes that patients who received transitional medical care are more likely to undergo outpatient treatment visits. The risk of not performing a promised outpatient visit was approximately 0.704 [29]. <Table 2>

**Table 2:** Risk ratios by transition states

Transition states		Risk Ratio (Range)	Source
Not readmitted	Stable management of COPD	-	-
	Not managing COPD	0.704 (0.634–0.774)	[29]
Readmitted	COPD	0.599 (0.539–0.659)	[28]
	respiratory diseases	0.720 (0.648–0.792)	[28]
	other diseases	0.720 (0.648–0.792)	[28]

Considering the difference in the average severity by age group [17], cohorts by age group were formed by dividing the starting point of the model into 60, 70, and 80 years of age. Transition probabilities, treatment costs, and additional mortality risks were calculated for each cohort.

The baseline analysis results are expressed as incremental cost-utility ratio (ICUR) values, and both deterministic sensitivity analyses (DSA) and probabilistic sensitivity analyses (PSA) are performed to test the uncertainty of the baseline result. Tornado diagrams from the DSA indicated important contributors to the model’s results. For PSA, costs were varied according to a gamma distribution, utilities and transition probability by a beta distribution, and relative risks by a log-normal distribution. Cost-effectiveness acceptability curves (CEACs) were calculated using nonparametric bootstrap resampling with replacement with 10,000 iterations.

All calculations were conducted with Microsoft Excel Professional Plus 2019. To evaluate the validity of the model applied in this study, the Assessment of the Validation Status of Health-Economic decision models (AdviSHE) tool [30] was used. An additional file shows this in more detail [See Additional File 1].

For the mortality rate for the “COPD stable management” health condition, the 1-year complete life table mortality rate provided by the National Statistical Office [31] was used, and the mortality risk rate for other health conditions was calculated by multiplying the additional mortality risk ratio to the mortality rate for each age.

The additional mortality due to “COPD readmission” was 1.66 times per year [32], and the additional mortality due to “not managing COPD” was 2.34 times at three years and 1.328 times per year [33]. Additional mortality due to “readmission for respiratory diseases,” excluding COPD, was 1.14 times at 18 months and 1.091 times per year. When patients with COPD were hospitalized for diseases “other than respiratory diseases,” it was 1.18 times higher at 18 months and 1.117 times higher per year [34]. An additional file shows this in more detail [See Additional File 2]. Discounting was set to 4.5% per year for costs and outcomes in line with the guidelines for economic evaluation [35]. The half-cycle correction was applied to unadjusted model outputs of the Markov model [36].

### Cost inputs

The analyses were conducted from a healthcare system perspective, including reimbursement and non-reimbursement payments. According to the “Pharmaceutical Economic Evaluation Guidelines” published by the Health Insurance Review and Assessment Service, direct healthcare costs include medicines (therapeutic drugs and side effects treatment), medical treatment (outpatient) services, hospital (inpatient) services, diagnosis and examination, and extra healthcare costs which are spent within the boundary of the NHIS [35].

However, since non-reimbursement payments cannot be calculated from claims data, the amount of non-reimbursement payments was calculated by multiplying the average share of non-reimbursement (21.37%) announced by the Korean Tuberculosis and Respiratory Society [37].

The nursing fee was estimated using the cost of care for the Integrated Nursing Care Service (INCS), in which professional nursing personnel in the hospital take care of patients instead of the patients’ families or paid caregivers. The average nursing fee was calculated by multiplying the INCS cost per day (85.82 USD, KRW 111,700) [38] and the average number of hospitalization days [39]. However, it was assumed that there was no difference in daily care costs according to age. In addition, the cost of regularly prescribed inhalers during outpatient visits was estimated by collecting the types of inhalers, applying the NHIS pharmaceutical benefit table [40] to confirm the cost of inhalers, and averaging the inhaler cost by age group. The sum of all medical costs is presented in Table 3.

**Table 3:** Total medical cost by age cohort and transition states

Cohort	Transition states		Total medical cost	Reimbursed costs	Not- reimbursed costs	Nursing fee	Prescribed Inhaler cost
60s	Not readmitted	Stable management of COPD	76.20				76.20
	Readmitted	COPD	2395.13	1245.09	266.05	883.99	
		respiratory diseases	2931.74	1906.45	407.36	617.93	
		other diseases	2976.15	1907.68	407.63	660.85	
70s	Not readmitted	Stable management of COPD	100.18				100.18
	Readmitted	COPD	2467.40	1304.64	278.77	883.99	
		respiratory diseases	3078.42	2027.30	433.18	617.93	
		other diseases	2933.76	1872.75	400.16	660.85	
80s	Not readmitted	Stable management of COPD	98.09				98.09
	Readmitted	COPD	2556.49	1378.05	294.46	883.99	
		respiratory diseases	3147.07	2083.87	445.27	617.93	
		other diseases	2822.57	1781.14	380.58	660.85	

* 1 USD = 1,301.50 KRW

Information on the per-patient cost of TCS was collected through interviews with nurses who is conducting the TC in South Korea. The main content of the interviews concerned how to implement the TCS and the time required. Based on this information, the time required by the coordinator nurse to manage one patient with COPD was calculated and is presented in <Table 4>.

**Table 4:** TCS cost per-patient by intervention

Interventions	Hours or fixed cost	Cost(USD $)
Screening	0.5 h	7.68
Ward visit– Baseline evaluation and EMR confirmation	2 h	30.73
Record evaluation result and set Care plan	0.5 h	7.68
Education	1 h	15.37
Ward visit– build rapport	10 min * 3 times	7.68
Home visit (within 48 h after discharge)	1.5 h	23.05
Phone call visit (once a week, 4 weeks)	20 min * 4 times	20.44
Home visit– Post evaluation and EMR confirmation (after one month)	1.5 h	23.05
Hospital visit - Follow-up evaluation and EMR confirmation (after three month)	1.5 h	23.05
Business trip expense (Home visits: within 48 h and after one month)	46.26 USD * 2 times	92.52
	Total	251.25

### Utility inputs

The European Quality of Life Five Dimensions (EQ-5D) is a standardized instrument that measures health-related quality of life [41] and allows the calculation of a quality-adjusted life year (QALY) value. A patient’s quality of life is assumed to depend on their transition state at the end of every cycle. Therefore, we assigned QALY values to the five transitional states, as suggested in prior studies.

Menn et al. have derived the utility of patients with COPD when hospitalized and discharged [23]. Furthermore, Szende has estimated the utility of patients hospitalized for respiratory (asthma) diseases [42]. Finally, Lin et al. have estimated the utility of hospitalizing patients in an acute care ward [43]. The utility values for each transition states were considered the same, regardless of the patient’s age. <Table 5>.

**Table 5:** Utilities by transition states

Transition states		Utility (Range)	Source
Not readmitted	Stable management of COPD	0.795 (0.716–0.875)	[23]
	Not managing COPD	0.795 (0.716–0.875)	[23]
Readmitted	COPD	0.61 (0.549–0.671)	[23]
	respiratory diseases	0.52 (0.468–0.572)	[42]
	other diseases	0.440 (0.396–0.484)	[43]

## Results

### Baseline results

A cost-benefit analysis showed that the implementation of the TCS was cost-effective. To a group that participated in the TCS, the total cost is reduced by 225.16 USD for those in their 60s, 1668 USD for those in their 70s, and 2251.64 USD for those in their 80s compared to those who did not participate. In addition, the QALY increased when TCS was performed in all age groups, by 0.231, 0.275, and 0.296 QALY for those in their 60s, 70s, and 80s, respectively, and the size of the effect increased with age. Combining these elements and calculating the Incremental Cost-Utility Ratio (ICUR), we found it in the negative range, implying that conducting TCS was a cost-saving strategy. <Table 6>.

**Table 6:** Baseline result of cost-utility analysis

Age cohort	Arm	Total cost (USD)	Diff. in cost (ΔUSD)	Total effect (QALYs)	Diff. in effect (ΔQALYs)	ICUR (USD/QALY)
60s	TCS	30123.92	-225.16	5.688	0.231	Cost-saving
	Usual care	30349.08		5.458		
70s	TCS	33076.24	-1668.00	5.293	0.275	Cost-saving
	Usual care	34744.24		5.017		
80s	TCS	29635.70	-2251.64	4.230	0.296	Cost-saving
	Usual care	31887.35		3.934		

### Assessment of uncertainty

A DSA was performed to evaluate the uncertainty by changing the discount rate, relative risk, transition probability, TCS cost, and treatment cost, which were input into the baseline analysis result. [See Additional File 3] In all age groups, TCS cost had the most significant influence on the basic analysis results of the ICUR, followed by the relative risk or cost of readmission for other diseases. [See Additional File 4].

**Fig. 2:**
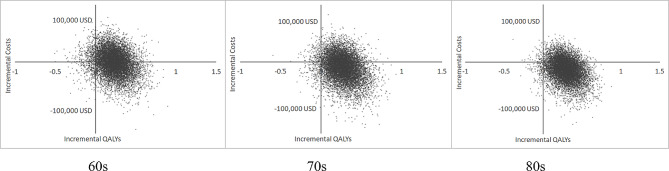
ICUR scatter plot by age

**Fig. 3:**
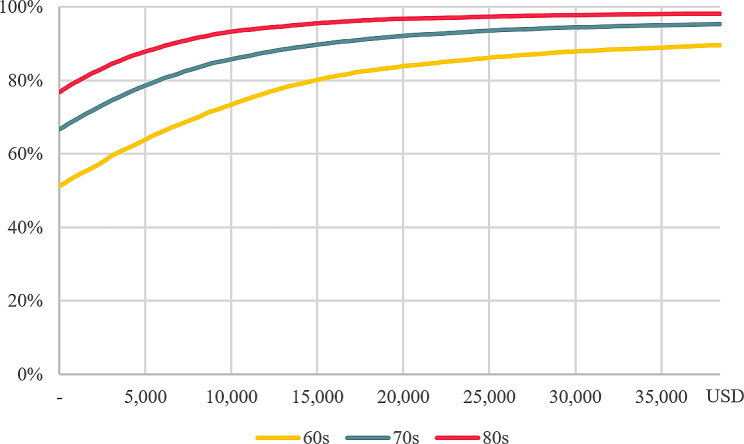
Cost-effective acceptability curves (CEAC) by age cohort

For PSA, a Monte Carlo simulation with 10,000 repetitions was performed. From the ICUR scatter plot by age, it was found that the higher the age, the stronger the centrality and the move toward the fourth quadrant. <Figure 2 > In addition, CEACs show that there is a 93.9%, 96.4%, and 98.2% probability for those in their 60s, 70s, and 80s, respectively, that TCS is cost-effective at a WTP threshold of 23,050 USD (KRW 30 million) per QALY. <Fig. 3>.

## Discussion

### Interpretation of results

This study analyzed the cost-effectiveness of TCS in patients with COPD according to age. As a result of this study, implementing TCS is a dominant alternative because medical costs decrease, and the QALY increases when implementing TCS [44]. Medical costs are low despite the implementation of TCS because medical use, such as hospitalization, is reduced when implementing TCS.

In contrast, the QALY was higher in the TCS group than in the non-TCS group. This result is meaningful because it reflects the results of a previous studies that a discharge patient management program would help patients with chronic diseases live healthier lives and reduce medical costs [45], and improve quality of life [46].

The “three dimensions of continuity” [47–48] can explain its effects on the TCS. Continuity of care occurs when patients experience coherent, connected, and consistent healthcare events related to their complex care needs [47]. “Relational continuity” refers to the establishment of a continuous relationship between patients, family caregivers, and care providers [48]. A continuous patient–provider relationship helps bridge discontinuous circumstances and gives patients and caregivers a sense of predictability and coherence [47]. In a study that provided discharge planning interventions for older patients with respiratory diseases who visited the emergency room, the patients showed a higher need for social support than younger groups to be satisfied with the nurses’ visits and caregiving [49]. Likewise, nurses can build a rapport with patients when providing TCS, and the strength of interpersonal relationships may influence patients’ compliance with TCS.

“Informational continuity” refers to the transfer and use of information from previous events and conditions to plan appropriate interventions [48]. While providing TCS, nurses collect comprehensive information regarding the patients, establish customized intervention strategies based on the collected information, continuously monitor self-management implementation, and help patients receive the necessary medical or welfare services. Additionally, TCS interventions modify the care plan according to the patients’ needs and care events.

Finally, TCS demonstrates “management continuity,” which refers to the consistent and timely coordination of care and services [48]. Under usual care, most patients are discharged with potential risk factors that may cause readmission [50]. For example, over half of the patients need help to use inhalers properly [51]. Moreover, 93.4% of the patients unfamiliar with inhalers preferred in-person education by medical staff when learning how to use them. However, 53.7% of pulmonologists responded that they lacked the time and staff to provide in-person education [12]. Furthermore, TCS include educational sessions regarding the use of inhalers, breathing techniques, symptom relief therapy in cases of acute exacerbation, exercise and management plans, and other disease management plans. In addition, counseling and re-education are provided to patients who fail to maintain self-management due to memory decline caused by aging or lack of motivation.

Furthermore, the probability of hospitalization for diseases other than COPD significantly influenced the economic effect of TCS. COPD is a systemic disease that affects the entire body and includes unintentional weight loss, skeletal muscle dysfunction, cardiovascular disease, osteoporosis, and depression [52]. Therefore, when implementing TCS, researchers must develop management strategies for patients with underlying diseases or frequent COPD comorbidities.

### Comparison with other studies

This study referred to a series of services that established a care plan for discharging patients, conducted the necessary education, and conducted home visits or phone checks as TCS. However, prior researchers have named these services variously and conducted similar economic evaluations.

In a meta-analysis study of the Hospital Readmissions Reduction Program conducted by the US Center for Medicare and Medicaid, the odds ratios for readmission and emergency room visits within 30 days after discharge were statistically significant as 0.73 (95% CI:0.54–0.98) and 0.67 (95% CI:0.50–0.88) respectively [53].

Similar to this study’s results, previous studies also predicted a total medical cost reduction due to reduced readmissions when a discharged patient management program was implemented. A study on the re-engineered Discharge program conducted by the Agency for Healthcare Research and Quality in the United States also shows that approximately $412 of medical expenses were saved for the patients who participated in the intervention [46]. Furthermore, a study by Charles Yan (2023) outlines that, despite the cost of CAN$ 308 per person when providing a care bundle to discharged patients, the medical care of patients enrolled in the care bundle cost less (TCB: CAN$ 10,172) than that of daily care (CAN$ 15,588) [18].

In the case of partial implementation of the discharge patient management program, the results regarding its effectiveness were mixed. Telephone monitoring [54], smoking cessation programs [24], and pulmonary rehabilitation maintenance education programs [55] have been reported to be cost-effective. However, in the case of providing medication reconciliation, primary care provider communication [56], community care, self-management promotion education, and medication adherence [21], the intervention effect was found to be neither different nor more expensive than routine treatment. Therefore, it seems desirable that a management program for discharged patients be provided in an integrated manner rather than a fragmented one.

### Limitations

It is possible that the TCS economic evaluation results were overestimated because of underestimated indirect costs. For example, the costs of protocol development for TCS, training coordinator nurses, and space in hospitals were not considered in this study. These costs may be decreased per patient as the number of patients participating in the program increases, according to the “economies of scale” law. Nonetheless, these costs can act as a practical entry barrier from the perspective of medical institutions, as high costs must be invested in the initial stage. To provide policymakers with accurate cost-effectiveness information on TCS, future analyses that consider these costs are necessary.

Another limitation arises from the input values of the model, as results from prior research were used to calculate cost-effectiveness. Because there is no information on the relative risk and utility values for each transition state of patients with COPD in Korea, the results of previous studies were used. Therefore, the actual results differ owing to differences between the interventions, medical service systems, and patient characteristics of Korea and those in previous studies. The responses of South Koreans should be considered in future research.

Finally, a previous study has reported a gradual decrease in inhaler compliance in patients with pulmonary disease [57]. We could not consider this because of the lack of previous studies on patient compliance, repetitive learning effects, or reduced compliance with discharge patient management programs. Developing and applying a more advanced methodology, including temporal changes in patient inhaler compliance, through a longitudinal analysis of discharged patients, is necessary.

Despite these limitations, this study is significant because a) it evaluated the long-term cost-effectiveness of TCS based on Korean claims data; 2) included possible medical use states as the transition states of the Markov model; and 3) applied the risk ratios to each transition states assuming that the effect of TCS varies by the given circumstances. Furthermore, by dividing the starting point of the cohort into people in their 60s, 70s, and 80s, this study presents evidence regarding the age groups at which TCS can be cost-effective.

## Conclusion

This study evaluated the economic feasibility of implementing a discharge management program for patients with COPD. TCS appeared to be a superior alternative to usual care because the program reduced readmissions and resulted in greater healthcare cost savings than the cost of implementing the program. However, for those in their 60s, cost-effectiveness varied greatly depending on the relative risk of TCS cost and other diseases; therefore, it is advantageous to preferentially consider patients aged 70 years or older with severe symptoms for TCS. In addition, it is important to include the management of underlying conditions and comorbidities in TCS.

The field of health medicine has recently undergone a paradigm shift from treatment to prevention and management [58]. TCS prevent readmission in discharged patients. The proportion of medical expenses due to readmission to the total cost of COPD is about 35–45% [53]; therefore, efforts to reduce readmission are necessary. This study provides evidence that the comprehensive management of discharged patients can improve their quality of life and reduce preventable and unnecessary medical expenses.

### Electronic supplementary material

Below is the link to the electronic supplementary material.

Supplementary Material 1

Supplementary Material 2

Supplementary Material 3

Supplementary Material 4

## Data Availability

Data available on request.
