# Supplementary material for: Cost-utility analysis of transitional care services for older inpatients with chronic obstructive pulmonary disease (COPD) in Korea
Source: Cost Eff Resour Alloc. 2024 Mar 2;22:19. doi: 10.1186/s12962-024-00526-3 (PMC10908012; doi:10.1186/s12962-024-00526-3)
Supplement: Supplementary file 1 — Supplementary Material 1 [file 12962_2024_526_MOESM1_ESM.docx]

Appendix Table 1. AdviSHE (Assessment of the Validation Status of Health-Economic decision models)

| **A. Validation of conceptual model** | | | |
| --- | --- | --- | --- |
| A1 | Face validity testing  (conceptual model) | Have experts been asked to judge  the appropriateness of the conceptual model? | Yes |
| A2 | Cross validity | Has this model been compared to  other conceptual models found in the literature or clinical textbooks? | Yes |
| **B. Input data validation** | | | |
| B1 | Face validity testing  (input data) | Have experts been asked to judge the appropriateness of the input data? | Yes |
| B2 | Model fit testing | When input parameters are based on regression models, have  statistical tests been performed? | No (Not applicable) |
| **C. Validation of computerized model** | | | |
| C1 | External review | Has the computerized model been examined by modelling experts? | Yes |
| C2 | Extreme value testing | Has the model been run for specific, extreme sets of  parameter values in order to detect any coding errors? | Yes |
| C3 | Testing of traces | Have patients been tracked through the model to determine  whether its logic is correct? | No (Not applicable) |
| C4 | Unit Testing | Have individual sub-modules of the computerized model been tested? | Yes |
| **D. Operational Validation** | | | |
| D1 | Face validity testing  (model outcomes) | Have experts been asked to judge the appropriateness of the model outcomes? | Yes |
| D2 | Cross validation testing  (model outcomes) | Have the model outcomes been  compared to the outcomes of other models that address similar problems? | Yes |
| D3 | Validation against  outcomes using  alternative input data | Have the model  outcomes been compared to the outcomes obtained when using alternative input data? | Yes |
| D4 | Validation against  empirical data | Have the model outcomes been compared to  empirical data?  D4.A: Comparison against the data sources on which the model is based  D4.B: Comparison against a data source that was not used to build the model | D4.A: Yes  D4.B: Yes |
| **E. Other validation technique** | | | |
| E1 | Other validation technique | Have any other validation techniques been performed? | No |
