# Supplementary material for: Cost-utility analysis of transitional care services for older inpatients with chronic obstructive pulmonary disease (COPD) in Korea
Source: Cost Eff Resour Alloc. 2024 Mar 2;22:19. doi: 10.1186/s12962-024-00526-3 (PMC10908012; doi:10.1186/s12962-024-00526-3)
Supplement: Supplementary file 2 — Supplementary Material 2 [file 12962_2024_526_MOESM2_ESM.docx]

Appendix Table 2. Mortality rate by age and transition status

| Age | All-cause  Mortality  rate1) | Mortality rate of  COPD readmission2) | | Mortality rate of  no management of  COPD2) | | Mortality rate of  respiratory disease | | Mortality rate of  other disease | |
| --- | --- | --- | --- | --- | --- | --- | --- | --- | --- |
|  |  | rate/year | probability/  year | rate/year | probability/  year | rate/year | probability/  year | rate/year | probability/  year |
| 60 | 0.00458 | 0.00762 | 0.00759 | 0.00609 | 0.00608 | 0.00501 | 0.00500 | 0.00513 | 0.00511 |
| 61 | 0.00491 | 0.00817 | 0.00814 | 0.00653 | 0.00651 | 0.00537 | 0.00536 | 0.00550 | 0.00548 |
| 62 | 0.00530 | 0.00882 | 0.00878 | 0.00705 | 0.00703 | 0.00580 | 0.00578 | 0.00593 | 0.00592 |
| 63 | 0.00575 | 0.00957 | 0.00953 | 0.00766 | 0.00763 | 0.00629 | 0.00627 | 0.00644 | 0.00642 |
| 64 | 0.00624 | 0.01039 | 0.01034 | 0.00831 | 0.00828 | 0.00683 | 0.00681 | 0.00699 | 0.00697 |
| 65 | 0.00679 | 0.01131 | 0.01125 | 0.00905 | 0.00900 | 0.00743 | 0.00741 | 0.00761 | 0.00758 |
| 66 | 0.00746 | 0.01243 | 0.01235 | 0.00994 | 0.00989 | 0.00817 | 0.00814 | 0.00836 | 0.00833 |
| 67 | 0.00827 | 0.01379 | 0.01369 | 0.01102 | 0.01096 | 0.00906 | 0.00902 | 0.00927 | 0.00923 |
| 68 | 0.00912 | 0.01521 | 0.01509 | 0.01216 | 0.01209 | 0.01000 | 0.00995 | 0.01023 | 0.01018 |
| 69 | 0.01009 | 0.01683 | 0.01669 | 0.01346 | 0.01337 | 0.01107 | 0.01101 | 0.01132 | 0.01126 |
| 70 | 0.01107 | 0.01848 | 0.01831 | 0.01478 | 0.01467 | 0.01215 | 0.01207 | 0.01243 | 0.01235 |
| 71 | 0.01223 | 0.02043 | 0.02022 | 0.01634 | 0.01620 | 0.01343 | 0.01334 | 0.01374 | 0.01365 |
| 72 | 0.01362 | 0.02276 | 0.02251 | 0.01821 | 0.01804 | 0.01496 | 0.01485 | 0.01531 | 0.01520 |
| 73 | 0.01535 | 0.02568 | 0.02535 | 0.02054 | 0.02033 | 0.01688 | 0.01674 | 0.01727 | 0.01713 |
| 74 | 0.01758 | 0.02944 | 0.02901 | 0.02355 | 0.02327 | 0.01935 | 0.01917 | 0.01981 | 0.01961 |
| 75 | 0.02017 | 0.03382 | 0.03326 | 0.02705 | 0.02669 | 0.02224 | 0.02199 | 0.02275 | 0.02250 |
| 76 | 0.02316 | 0.03890 | 0.03815 | 0.03111 | 0.03063 | 0.02557 | 0.02525 | 0.02617 | 0.02583 |
| 77 | 0.02652 | 0.04462 | 0.04364 | 0.03568 | 0.03505 | 0.02933 | 0.02890 | 0.03001 | 0.02957 |
| 78 | 0.03024 | 0.05097 | 0.04970 | 0.04077 | 0.03995 | 0.03351 | 0.03295 | 0.03429 | 0.03371 |
| 79 | 0.03455 | 0.05837 | 0.05670 | 0.04668 | 0.04561 | 0.03837 | 0.03764 | 0.03926 | 0.03850 |
| 80 | 0.03940 | 0.06673 | 0.06455 | 0.05337 | 0.05197 | 0.04386 | 0.04292 | 0.04489 | 0.04389 |
| 81 | 0.04507 | 0.07655 | 0.07370 | 0.06123 | 0.05939 | 0.05032 | 0.04908 | 0.05150 | 0.05019 |
| 82 | 0.05147 | 0.08772 | 0.08398 | 0.07015 | 0.06775 | 0.05766 | 0.05603 | 0.05901 | 0.05730 |
| 83 | 0.05859 | 0.10023 | 0.09537 | 0.08016 | 0.07703 | 0.06588 | 0.06376 | 0.06742 | 0.06520 |
| 84 | 0.06658 | 0.11437 | 0.10808 | 0.09147 | 0.08741 | 0.07519 | 0.07243 | 0.07694 | 0.07405 |
| 85 | 0.07524 | 0.12985 | 0.12177 | 0.10385 | 0.09864 | 0.08536 | 0.08182 | 0.08735 | 0.08364 |
| 86 | 0.08477 | 0.14704 | 0.13674 | 0.11760 | 0.11095 | 0.09666 | 0.09214 | 0.09891 | 0.09418 |
| 87 | 0.09522 | 0.16611 | 0.15304 | 0.13284 | 0.12440 | 0.10919 | 0.10344 | 0.11174 | 0.10572 |
| 88 | 0.10662 | 0.18715 | 0.17068 | 0.14968 | 0.13901 | 0.12303 | 0.11576 | 0.12590 | 0.11829 |
| 89 | 0.11902 | 0.21036 | 0.18970 | 0.16823 | 0.15484 | 0.13828 | 0.12915 | 0.14150 | 0.13195 |
| 90 | 0.13245 | 0.23586 | 0.21011 | 0.18863 | 0.17191 | 0.15504 | 0.14362 | 0.15866 | 0.14671 |
