# Supplementary material for: Cost-utility analysis of transitional care services for older inpatients with chronic obstructive pulmonary disease (COPD) in Korea
Source: Cost Eff Resour Alloc. 2024 Mar 2;22:19. doi: 10.1186/s12962-024-00526-3 (PMC10908012; doi:10.1186/s12962-024-00526-3)
Supplement: Supplementary file 3 — Supplementary Material 3 [file 12962_2024_526_MOESM3_ESM.docx]

Appendix Table 3. Input parameters for baseline analysis and sensitivity analyses (60s)

| **Input parameter** | **Baseline** | **DSA** | | **PSA** | **Sources** |
| --- | --- | --- | --- | --- | --- |
|  |  | **Low** | **High** |  |  |
| **Discount rate(cost, effect)** | 4.5% | 3.5% | 6.0% | - | [93] |
| **Transition probability** |  |  |  |  |  |
| COPD readmission | 0.0748 | 0.0673 | 0.0823 | Beta (α = 14.7; β = 182.2) | NHIS-SC |
| Stable management of COPD | 1 – other transition probabilities | | | - | - |
| No management of COPD | 0.3289 | 0.2960 | 0.3618 | Beta (α = 10.4; β = 21.2) | NHIS-SC |
| Respiratory disease readmission | 0.0684 | 0.0616 | 0.0752 | Beta (α = 14.8; β = 202.1) | NHIS-SC |
| Other disease readmission | 0.1806 | 0.1625 | 0.1987 | Beta (α = 12.9; β = 58.7) | NHIS-SC |
| Death | Mortality rate (yearly) by age * additional mortality rate by transition status | | | | [90-92] |
| **Risk Ratios** |  |  |  |  |  |
| COPD readmission | 0.599 | 0.539 | 0.659 | Normal (LN(avg) –0.512 LN(s.d) 0.1127) | [85] |
| No management of COPD | 0.704 | 0.634 | 0.774 | Normal (LN(avg) –0.329 LN(s.d) 0.1127) | [86] |
| Respiratory disease readmission | 0.720 | 0.648 | 0.792 | Normal (LN(avg) –0.329 LN(s.d) 0.1127) | [85] |
| Other disease readmission | 0.720 | 0.648 | 0.792 | Normal (LN(avg) –0.351 LN(s.d) 0.1127) | [85] |
| **Cost** |  |  |  |  |  |
| TCS intervention cost | 327,000 | 245,250 | 408,750 | Gamma (α= 16.0; β= 20437.5) | RCT |
| COPD readmission | 3,117,259 | 2,337,944 | 3,896,573 | Gamma (α= 16.0; β= 194828.7) | NHIS-SC, [77-81] |
| No management of COPD | 0 | 0 | 0 |  |  |
| Stable management of COPD | 99,176 | 74,382 | 123,970 | Gamma (α= 16.0; β= 6198.5) | NHIS-SC, [77-81] |
| Respiratory disease readmission | 3,815,662 | 2,861,746 | 4,769,577 | Gamma (α= 16.0; β= 238478.9) | NHIS-SC, [77-81] |
| Other disease readmission | 3,873,464 | 2,905,098 | 4,841,829 | Gamma (α= 16.0; β= 242091.5) | NHIS-SC, [77-81] |
| **Utility** |  |  |  |  |  |
| COPD readmission | 0.610 | 0.549 | 0.671 | Beta (α = 38.4; β = 24.5) | [82] |
| Stable management of COPD | 0.795 | 0.716 | 0.875 | Beta (α = 19.7; β = 5.1) | [82] |
| No management of COPD | 0.795 | 0.716 | 0.875 | Beta (α = 19.7; β = 5.1) | [82] |
| Respiratory disease readmission | 0.520 | 0.468 | 0.572 | Beta (α = 47.5; β = 43.8) | [83] |
| Other disease readmission | 0.440 | 0.396 | 0.484 | Beta (α = 55.6; β = 70.7) | [84] |

** NHIS-SC* National Health Insurance Service, *DSA* Deterministic sensitivity analysis, *PSA* Probabilistic sensitivity analysis, *avg* average, *s.d* standard deviation

* For indicators with unknown confidence intervals in sensitivity analysis, ±10% for ratios and ±25% for costs are applied.

Appendix Table 4. Input parameters for baseline analysis and sensitivity analyses (70s)

| **Input parameter** | **Baseline** | **DSA** | | **PSA** | **Sources** |
| --- | --- | --- | --- | --- | --- |
|  |  | **Low** | **High** |  |  |
| **Discount rate(cost, effect)** | 4.5% | 3.5% | 6.0% | - | [93] |
| **Transition probability** |  |  |  |  |  |
| COPD readmission | 0.0748 | 0.0673 | 0.0823 | Beta (α = 14.6; β = 161.0) | NHIS-SC |
| Stable management of COPD | 1 – other transition probabilities | | | - | - |
| No management of COPD | 0.3289 | 0.2930 | 0.3618 | Beta (α = 10.8; β = 24.9) | NHIS-SC |
| Respiratory disease readmission | 0.0684 | 0.0616 | 0.0753 | Beta (α = 14.6; β = 156.9) | NHIS-SC |
| Other disease readmission | 0.1806 | 0.1625 | 0.1987 | Beta (α = 12.3; β = 43.5) | NHIS-SC |
| Death | Mortality rate (yearly) by age * additional mortality rate by transition status | | | | [90-92] |
| **Risk Ratios** |  |  |  |  |  |
| COPD readmission | 0.599 | 0.539 | 0.659 | Normal (LN(avg) –0.512 LN(s.d) 0.1127) | [85] |
| No management of COPD | 0.704 | 0.634 | 0.774 | Normal (LN(avg) –0.329 LN(s.d) 0.1127) | [86] |
| Respiratory disease readmission | 0.720 | 0.648 | 0.792 | Normal (LN(avg) –0.329 LN(s.d) 0.1127) | [85] |
| Other disease readmission | 0.720 | 0.648 | 0.792 | Normal (LN(avg) –0.351 LN(s.d) 0.1127) | [85] |
| **Cost** |  |  |  |  |  |
| TCS intervention cost | 327,000 | 245,250 | 408,750 | Gamma (α= 16.0; β= 20437.5) | RCT |
| COPD readmission | 3,117,259 | 2,337,944 | 3,896,573 | Gamma (α= 16.0; β= 200707.4) | NHIS-SC, [77-81] |
| No management of COPD | 0 | 0 | 0 |  |  |
| Stable management of COPD | 99,176 | 74,382 | 123,970 | Gamma (α= 16.0; β= 8149.3) | NHIS-SC, [77-81] |
| Respiratory disease readmission | 3,815,662 | 2,861,746 | 4,769,577 | Gamma (α= 16.0; β= 250410.3) | NHIS-SC, [77-81] |
| Other disease readmission | 3,873,464 | 2,905,098 | 4,841,829 | Gamma (α= 16.0; β= 238642.8) | NHIS-SC, [77-81] |
| **Utility** |  |  |  |  |  |
| COPD readmission | 0.610 | 0.549 | 0.671 | Beta (α = 38.4; β = 24.5) | [82] |
| Stable management of COPD | 0.795 | 0.716 | 0.875 | Beta (α = 19.7; β = 5.1) | [82] |
| No management of COPD | 0.795 | 0.716 | 0.875 | Beta (α = 19.7; β = 5.1) | [82] |
| Respiratory disease readmission | 0.520 | 0.468 | 0.572 | Beta (α = 47.5; β = 43.8) | [83] |
| Other disease readmission | 0.440 | 0.396 | 0.484 | Beta (α = 55.6; β = 70.7) | [84] |

** NHIS-SC* National Health Insurance Service, *DSA* Deterministic sensitivity analysis, *PSA* Probabilistic sensitivity analysis, *avg* average, *s.d* standard deviation

* For indicators with unknown confidence intervals in sensitivity analysis, ±10% for ratios and ±25% for costs are applied.

Appendix Table 5. Input parameters for baseline analysis and sensitivity analyses (80s)

| **Input parameter** | **Baseline** | **DSA** | | **PSA** | **Sources** |
| --- | --- | --- | --- | --- | --- |
|  |  | **Low** | **High** |  |  |
| **Discount rate(cost, effect)** | 4.5% | 3.5% | 6.0% | - | [93] |
| **Transition probability** |  |  |  |  |  |
| COPD readmission | 0.0748 | 0.0673 | 0.0823 | Beta (α = 14.3; β = 124.2) | NHIS-SC |
| Stable management of COPD | 1 – other transition probabilities | | | - | - |
| No management of COPD | 0.3289 | 0.2930 | 0.3618 | Beta (α = 10.7; β = 23.6) | NHIS-SC |
| Respiratory disease readmission | 0.0684 | 0.0616 | 0.0753 | Beta (α = 14.3; β = 127.0) | NHIS-SC |
| Other disease readmission | 0.1806 | 0.1625 | 0.1987 | Beta (α = 11.9; β = 37.5) | NHIS-SC |
| Death | Mortality rate (yearly) by age * additional mortality rate by transition status | | | | [90-92] |
| **Risk Ratios** |  |  |  |  |  |
| COPD readmission | 0.599 | 0.539 | 0.659 | Normal (LN(avg) –0.512 LN(s.d) 0.1127) | [85] |
| No management of COPD | 0.704 | 0.634 | 0.774 | Normal (LN(avg) –0.329 LN(s.d) 0.1127) | [86] |
| Respiratory disease readmission | 0.720 | 0.648 | 0.792 | Normal (LN(avg) –0.329 LN(s.d) 0.1127) | [85] |
| Other disease readmission | 0.720 | 0.648 | 0.792 | Normal (LN(avg) –0.351 LN(s.d) 0.1127) | [85] |
| **Cost** |  |  |  |  |  |
| TCS intervention cost | 327,000 | 245,250 | 408,750 | Gamma (α= 16.0; β= 20437.5) | RCT |
| COPD readmission | 3,117,259 | 2,337,944 | 3,896,573 | Gamma (α= 16.0; β= 207954.8) | NHIS-SC, [77-81] |
| No management of COPD | 0 | 0 | 0 |  |  |
| Stable management of COPD | 99,176 | 74,382 | 123,970 | Gamma (α= 16.0; β= 7979.1) | NHIS-SC, [77-81] |
| Respiratory disease readmission | 3,815,662 | 2,861,746 | 4,769,577 | Gamma (α= 16.0; β= 255994.4) | NHIS-SC, [77-81] |
| Other disease readmission | 3,873,464 | 2,905,098 | 4,841,829 | Gamma (α= 16.0; β= 229598.2) | NHIS-SC, [77-81] |
| **Utility** |  |  |  |  |  |
| COPD readmission | 0.610 | 0.549 | 0.671 | Beta (α = 38.4; β = 24.5) | [82] |
| Stable management of COPD | 0.795 | 0.716 | 0.875 | Beta (α = 19.7; β = 5.1) | [82] |
| No management of COPD | 0.795 | 0.716 | 0.875 | Beta (α = 19.7; β = 5.1) | [82] |
| Respiratory disease readmission | 0.520 | 0.468 | 0.572 | Beta (α = 47.5; β = 43.8) | [83] |
| Other disease readmission | 0.440 | 0.396 | 0.484 | Beta (α = 55.6; β = 70.7) | [84] |

** NHIS-SC* National Health Insurance Service, *DSA* Deterministic sensitivity analysis, *PSA* Probabilistic sensitivity analysis, *avg* average, *s.d* standard deviation

* For indicators with unknown confidence intervals in sensitivity analysis, ±10% for ratios and ±25% for costs are applied.
