# Supplementary material for: Cost-utility analysis of transitional care services for older inpatients with chronic obstructive pulmonary disease (COPD) in Korea
Source: Cost Eff Resour Alloc. 2024 Mar 2;22:19. doi: 10.1186/s12962-024-00526-3 (PMC10908012; doi:10.1186/s12962-024-00526-3)
Supplement: Supplementary file 4 — Supplementary Material 4 [file 12962_2024_526_MOESM4_ESM.docx]

Appendix Table 6. DSA Input and results of 60s

| Variables | Baseline  Input | DSA input | | ICUR (USD/QALY) | |
| --- | --- | --- | --- | --- | --- |
|  |  | Lower | Upper | Lower | Upper |
| Discount rate (effect) | 4.5% | 3.5% | 6.0% | -931.92 | -1042.62 |
| Discout rate (cost) | 4.5% | 3.5% | 6.0% | -1042.10 | -883.93 |
| RR of COPD readmission | 0.599 | 0.539 | 0.659 | -2291.54 | 420.60 |
| RR of resp. disease readmission | 0.720 | 0.648 | 0.792 | -2734.59 | 951.79 |
| RR of other disease readmission | 0.720 | 0.648 | 0.792 | -5214.57 | 4826.04 |
| RR of no management of COPD | 0.704 | 0.634 | 0.774 | -712.25 | -1241.31 |
| Prob. of COPD readmission | 0.075 | 0.067 | 0.082 | -53.28 | -1861.96 |
| Prob. of no management of COPD | 0.329 | 0.296 | 0.362 | -1086.08 | -865.52 |
| Prob. of resp. disease readmission | 0.068 | 0.062 | 0.075 | -251.07 | -1674.88 |
| Prob. of other disease readmission | 0.181 | 0.163 | 0.199 | 1041.41 | -2763.25 |
| Utility of COPD readmission | 0.610 | 0.549 | 0.671 | -919.39 | -1039.05 |
| Utility of no management of COPD | 0.795 | 0.716 | 0.875 | -775.40 | -1315.03 |
| Utility of stable management of COPD | 0.795 | 0.716 | 0.875 | -2060.96 | -639.02 |
| Utility of resp. disease readmission | 0.520 | 0.468 | 0.572 | -944.21 | -1009.07 |
| Utility of other disease readmission | 0.440 | 0.396 | 0.484 | -908.18 | -1053.74 |
| Cost of TCS intervention | 251.25 | 188.44 | 314.06 | -9516.56 | 7565.43 |
| Cost of COPD readmission | 2395.13 | 1796.35 | 2993.91 | 1423.64 | -3374.76 |
| Cost of stable management of COPD | 76.20 | 57.15 | 95.25 | -1480.36 | -470.77 |
| Cost of resp. disease readmission | 2931.74 | 2198.81 | 3664.68 | 896.67 | -2847.80 |
| Cost of other disease readmission | 2976.15 | 2232.12 | 3720.19 | 4042.68 | -5993.81 |
| *ICUR* Incremental Cost-Utility Ratio *RR* Relative Risk, *resp.* respiratory, *Prob* probability | | | | | |

Appendix Figure 2. Tornado diagram ICUR 60s

Appendix Figure 1. Tornado diagram ICUR 60s

Appendix Table 7. DSA Input and results of 70s

| Variables | Baseline  Input | DSA input | | ICUR (USD/QALY) | |
| --- | --- | --- | --- | --- | --- |
|  |  | Lower | Upper | Lower | Upper |
| Discount rate (effect) | 4.5% | 3.5% | 6.0% | -5788.31 | -6473.07 |
| Discout rate (cost) | 4.5% | 3.5% | 6.0% | -6341.68 | -5667.96 |
| RR of COPD readmission | 0.599 | 0.539 | 0.659 | -7089.45 | -4966.29 |
| RR of resp. disease readmission | 0.720 | 0.648 | 0.792 | -7652.08 | -4312.11 |
| RR of other disease readmission | 0.720 | 0.648 | 0.792 | -9408.53 | -1500.75 |
| RR of no management of COPD | 0.704 | 0.634 | 0.774 | -5724.59 | -6397.95 |
| Prob. of COPD readmission | 0.083 | 0.075 | 0.091 | -5340.83 | -6748.54 |
| Prob. of no management of COPD | 0.303 | 0.273 | 0.333 | -6197.98 | -5919.85 |
| Prob. of resp. disease readmission | 0.085 | 0.076 | 0.093 | -5407.73 | -6686.49 |
| Prob. of other disease readmission | 0.220 | 0.198 | 0.242 | -4484.72 | -7457.37 |
| Utility of COPD readmission | 0.610 | 0.549 | 0.671 | -5748.83 | -6403.06 |
| Utility of no management of COPD | 0.795 | 0.716 | 0.875 | -5098.51 | -7463.36 |
| Utility of stable management of COPD | 0.795 | 0.716 | 0.875 | -10939.18 | -4189.20 |
| Utility of resp. disease readmission | 0.520 | 0.468 | 0.572 | -5867.05 | -6262.50 |
| Utility of other disease readmission | 0.440 | 0.396 | 0.484 | -5654.15 | -6524.75 |
| Cost of TCS intervention | 251.25 | 188.44 | 314.06 | -12855.52 | 738.86 |
| Cost of COPD readmission | 2395.13 | 1796.35 | 2993.91 | -3880.66 | -8236.01 |
| Cost of stable management of COPD | 76.20 | 57.15 | 95.25 | -6620.59 | -5496.07 |
| Cost of resp. disease readmission | 2931.74 | 2198.81 | 3664.68 | -4128.28 | -7988.39 |
| Cost of other disease readmission | 2976.15 | 2232.12 | 3720.19 | -1292.03 | -10824.63 |
| *ICUR* Incremental Cost-Utility Ratio *RR* Relative Risk, *resp.* respiratory, *Prob* probability | | | | | |

Appendix Figure 2. Tornado diagram ICUR 70s

Appendix Table 8. DSA Input and results of 80s

| Variables | Baseline  Input | DSA input | | ICUR (USD/QALY) | |
| --- | --- | --- | --- | --- | --- |
|  |  | Lower | Upper | Lower | Upper |
| Discount rate (effect) | 4.5% | 3.5% | 6.0% | -7271.78 | -8100.30 |
| Discout rate (cost) | 4.5% | 3.5% | 6.0% | -7892.94 | -7190.62 |
| RR of COPD readmission | 0.599 | 0.539 | 0.659 | -8453.75 | -6682.22 |
| RR of resp. disease readmission | 0.720 | 0.648 | 0.792 | -9010.28 | -6061.37 |
| RR of other disease readmission | 0.720 | 0.648 | 0.792 | -10085.99 | -4358.13 |
| RR of no management of COPD | 0.704 | 0.634 | 0.774 | -7120.63 | -8097.69 |
| Prob. of COPD readmission | 0.103 | 0.093 | 0.113 | -7012.88 | -8157.30 |
| Prob. of no management of COPD | 0.312 | 0.280 | 0.343 | -7796.87 | -7404.92 |
| Prob. of resp. disease readmission | 0.101 | 0.091 | 0.111 | -7045.73 | -8134.62 |
| Prob. of other disease readmission | 0.241 | 0.217 | 0.265 | -6504.12 | -8590.82 |
| Utility of COPD readmission | 0.610 | 0.549 | 0.671 | -7239.83 | -7995.44 |
| Utility of no management of COPD | 0.795 | 0.716 | 0.875 | -6650.14 | -8863.42 |
| Utility of stable management of COPD | 0.795 | 0.716 | 0.875 | -12225.39 | -5512.71 |
| Utility of resp. disease readmission | 0.520 | 0.468 | 0.572 | -7387.89 | -7822.31 |
| Utility of other disease readmission | 0.440 | 0.396 | 0.484 | -7184.98 | -8063.42 |
| Cost of TCS intervention | 251.25 | 188.44 | 314.06 | -12734.89 | -2462.91 |
| Cost of COPD readmission | 2395.13 | 1796.35 | 2993.91 | -5520.34 | -9677.46 |
| Cost of stable management of COPD | 76.20 | 57.15 | 95.25 | -8065.83 | -7131.97 |
| Cost of resp. disease readmission | 2931.74 | 2198.81 | 3664.68 | -5870.37 | -9327.43 |
| Cost of other disease readmission | 2976.15 | 2232.12 | 3720.19 | -3903.34 | -11294.46 |
| *ICUR* Incremental Cost-Utility Ratio *RR* Relative Risk, *resp.* respiratory, *Prob* probability | | | | | |

Appendix Figure 3. Tornado diagram ICUR 80s
